# An Online Atlas for Exploring Spatio-Temporal Patterns of Cancer Mortality (1972–2011) and Incidence (1995–2008) in Taiwan

**DOI:** 10.1097/MD.0000000000003496

**Published:** 2016-05-27

**Authors:** Wen-Yuan Ku, Yung-Po Liaw, Jing-Yang Huang, Oswald Ndi Nfor, Shu-Yi Hsu, Pei-Chieh Ko, Wen-Chung Lee, Chien-Jen Chen

**Affiliations:** From the Department of Public Health and Institute of Public Health (W-YK, Y-PL, J-YH, ONN, S-YH, P-CK); Department of Family and Community Medicine (Y-PL), Chung Shan Medical University Hospital, Taichung City; Research Center for Genes (W-CL), Environment and Human Health, and Institute of Epidemiology and Preventive Medicine, College of Public Health, National Taiwan University; and Genomics Research Center (C-JC), Academia Sinica, Taipei, Taiwan.

## Abstract

Public health mapping and Geographical Information Systems (GIS) are already being used to locate the geographical spread of diseases. This study describes the construction of an easy-to-use online atlas of cancer mortality (1972–2011) and incidence (1995–2008) in Taiwan.

Two sets of color maps were made based on “age-adjusted mortality by rate” and “age-adjusted mortality by rank.” AJAX (Asynchronous JavaScript and XML), JSON (JavaScript Object Notation), and SVG (Scaling Vector Graphic) were used to create the online atlas. Spatio-temporal patterns of cancer mortality and incidence in Taiwan over the period from 1972 to 2011 and from 1995 to 2008.

The constructed online atlas contains information on cancer mortality and incidence (http://taiwancancermap.csmu-liawyp.tw/). The common GIS functions include zoom and pan and identity tools. Users can easily customize the maps to explore the spatio-temporal trends of cancer mortality and incidence using different devices (such as personal computers, mobile phone, or pad). This study suggests an easy- to-use, low-cost, and independent platform for exploring cancer incidence and mortality. It is expected to serve as a reference tool for cancer prevention and risk assessment.

This online atlas is a cheap and fast tool that integrates various cancer maps. Therefore, it can serve as a powerful tool that allows users to examine and compare spatio-temporal patterns of various maps. Furthermore, it is an-easy-to use tool for updating data and assessing risk factors of cancer in Taiwan.

## INTRODUCTION

Mapping spatial aspects of diseases is essential for understanding disease outbreak. Disease maps are important for health practitioners and the general population to visually communicate about disease distribution.^1^ Public health mapping and Geographical Information Systems (GIS) are already being used to locate the geographical spread of diseases in many countries.^2–7^ GIS is a powerful tool that can help epidemiologists to explore and analyze cancer data, hence generating ideas and hypotheses at the beginning of a research.^8^ Commercial GIS software had been used to construct cancer maps. However, some of the drawbacks include cost, inconvenience, limited platforms, and data update problems. With the improvement in web technology in recent years, the internet has become an important means to obtain information. In this study, we describe the construction and implementation of an easy to use, a low cost, and independent platform for exploring cancer incidence and mortality patterns in Taiwan.

## METHOD AND MATERIALS

The cancer mortality data (1972–2011) were collected from the national death databank of the Bureau of Health Promotion, Department of Health, Executive Yuan. Mortality data were divided into 4 periods (1972–1981, 1982–1991, 1992–2001, and 2002–2011). Incidence data (1995–2008) were collected from the Cancer Registry System (CRS), Bureau of Health Promotion, Department of Health, Executive Yuan. The incidence data were divided into 2 periods (1995–2001 and 2002–2009). Ethical approval and consents were not required for this study because it involved the analysis of deidentified data. Two sets of color maps were made based on the indices “age-standardized mortality/incidence rate (ASR)” and “age-standardized mortality/incidence rate by rank (*P*-value)” for both genders. The ASRs for mortality and incidence were examined by period, sex, cancer sites, and townships. The ASR for different townships was stratified by 18 age groups (0–4, 5–9… 80–84, and 85 and over), based on the 2000 World Standard Population. Cancer sites included the lip, oral cavity, major salivary glands, nasopharynx, esophagus, stomach, small intestine, colon, rectum, rectosigmoid junction and anus, liver and intrahepatic bile ducts, gallbladder and extrahepatic bile ducts, pancreas, nasal cavities, middle ear and accessory sinuses, larynx, trachea, bronchus and lung, bone and articular cartilage, connective and other soft tissues, skin (melanoma), skin (nonmelanoma), female breast, cervix uteri and uterus, parts unspecified, ovary and other uterine adnexa, prostate, bladder, kidney and other unspecified urinary organs, brain, thyroid gland, non-Hodgkin's lymphoma, leukemia, and all sites combined.

Maps were generated using the ASR and *P* values. Different colors (red–green gradients) were used to illustrate the 7 groups (levels 1–7) that were ranked by percentiles using the ASRs of the townships in Taiwan. The 7 levels represent the *P* values. The ASRs for each township were ranked from the highest to the lowest in all the townships. Townships ranked within the top 10 out of all townships in cancer mortality/incidence and for which the rates were significantly higher than the overall average in Taiwan were categorized into level 1 (red). Details of the rankings and comparisons of the township-specific ASRs with the overall ASR in Taiwan are shown in Table 1. Data were analyzed using the SAS statistical software version 9.3.

**TABLE 1:**
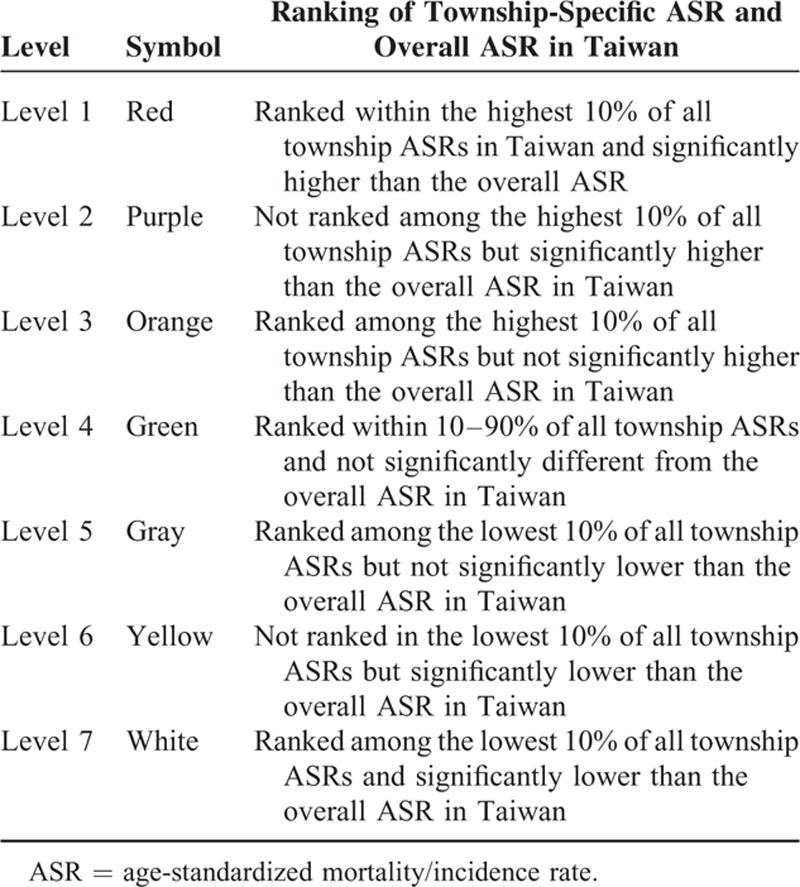
Age-Standardized Mortality and Incidence Rate by Rank for the 7 Groups (Levels 1–7) in Taiwan

This online atlas was constructed using AJAX (Asynchronous JavaScript and XML),^9^ JSON (JavaScript Object Notation),^10^ SVG (Scaling Vector Graphic),^11^ jQuery,^12^ and jVectorMap ^13^ technology. AJAX, described by Jesse James Garrett in 2005, incorporated a number of technologies for the front-end web development. AJAX is the data transfer technology, that is, the data information is passed from database to the browser which allows rapid change and exploration of cancer maps by gender or other indices. JSON is a lightweight data-interchange format when compared to XML. When obtaining cancer information through AJAX, JSON can reduce the amount of data transmission. jQuery is a lightweight JavaScript library which can easily control the browser actions such as zoom in, zoom out, pan, and query function. jVectorMap is a jQuery plugin that can be used to easily create interactive maps. Unlike the SVG, the GIS shapefile cannot be published on the web. Therefore, to publish the maps on the web, GIS data or shapefile ought to be converted into SVG format. jVectorMap can provide such a function as it uses SVG as the map format. It also has a Python converter for creating maps from the GIS shapefiles. With jVectorMap and SVG, cancer maps can have high resolution and interaction during exploration.

## RESULTS

**FIGURE 1:**
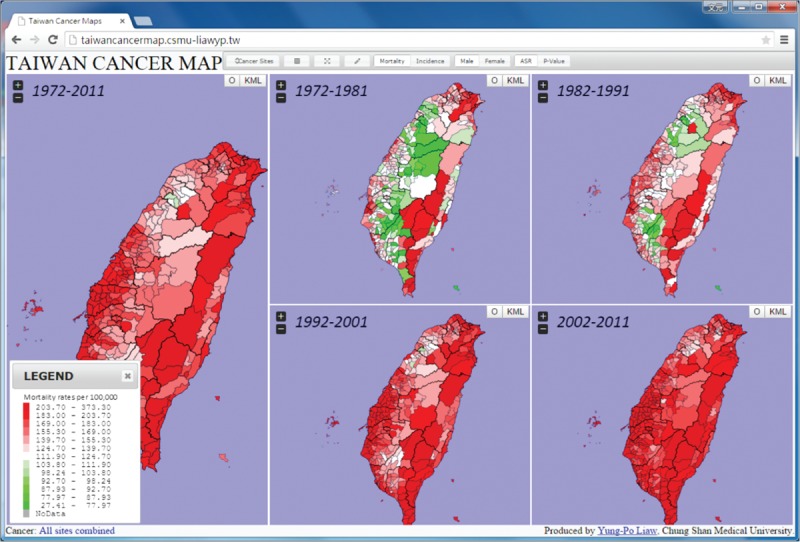
Age-standardized mortality rates for all townships 1972 to 2011 (per 100,000 persons).

**FIGURE 2:**
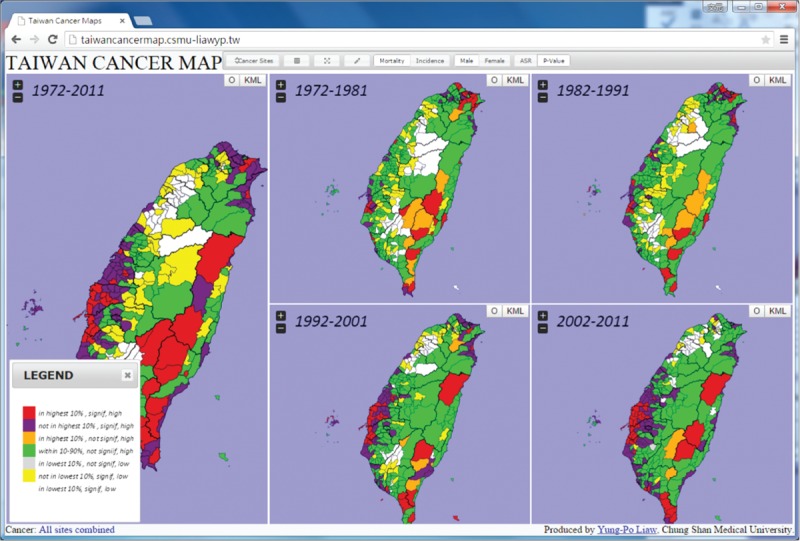
Ranks for the age-standardized mortality from 1972 to 2011.

**FIGURE 3:**
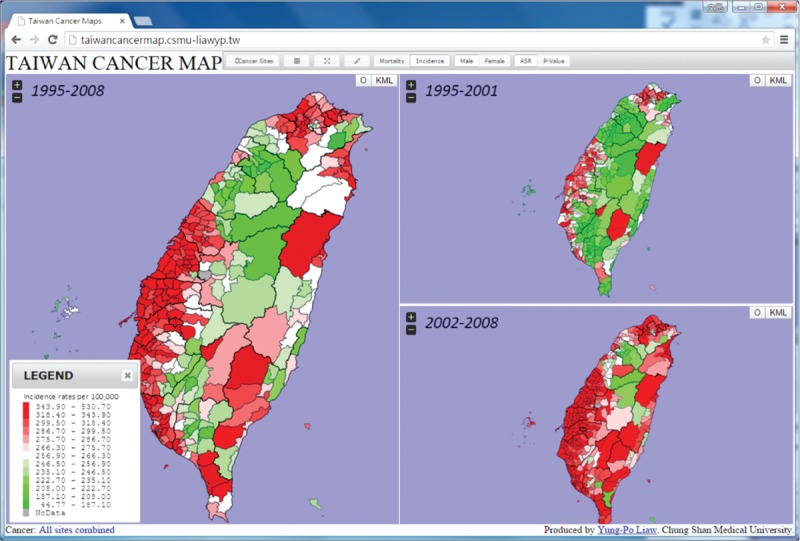
Age-standardized incidence rates for all townships, 1995 to 2008 (per 100,000 persons).

**FIGURE 4:**
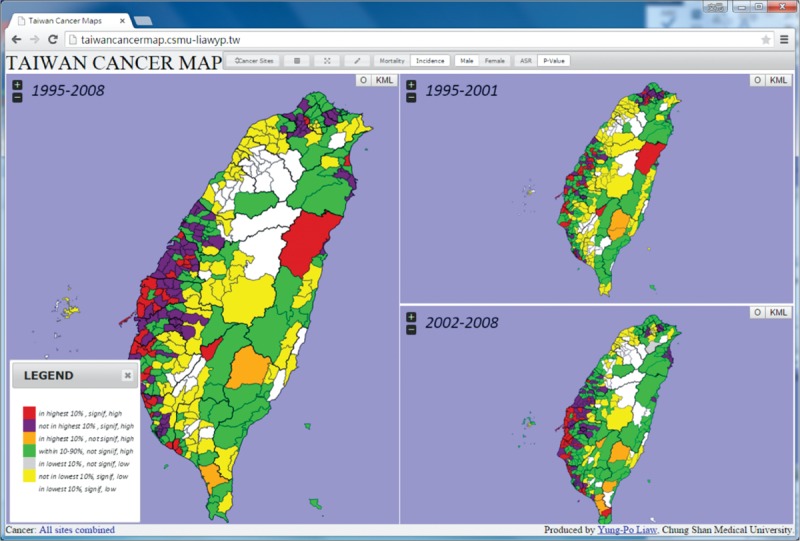
Ranks for the age-standardized incidence from 1995 to 2008.

In order to explore the online atlas, users have to browse the following link “http://taiwancancermap.csmu-liawyp.tw.” The age-standardized mortality rate is directly assessed for specific townships from 1972 to 2011 (1972–1981, 1982–1991, 1992–2001, and 2002–2011) as displayed in Figure 1. Clicking on the “select cancer” panel or icon (upper left corner) allows users to view cancer type by gender. Selecting the “Map Index” panel allows users to assess cancer by ASR or *P* value (Figure 2). The “Map type” panel is used to view the atlas by mortality or incidence (Figures 3 and 4 display incidence rates from 1995 to 2008).

**FIGURE 5:**
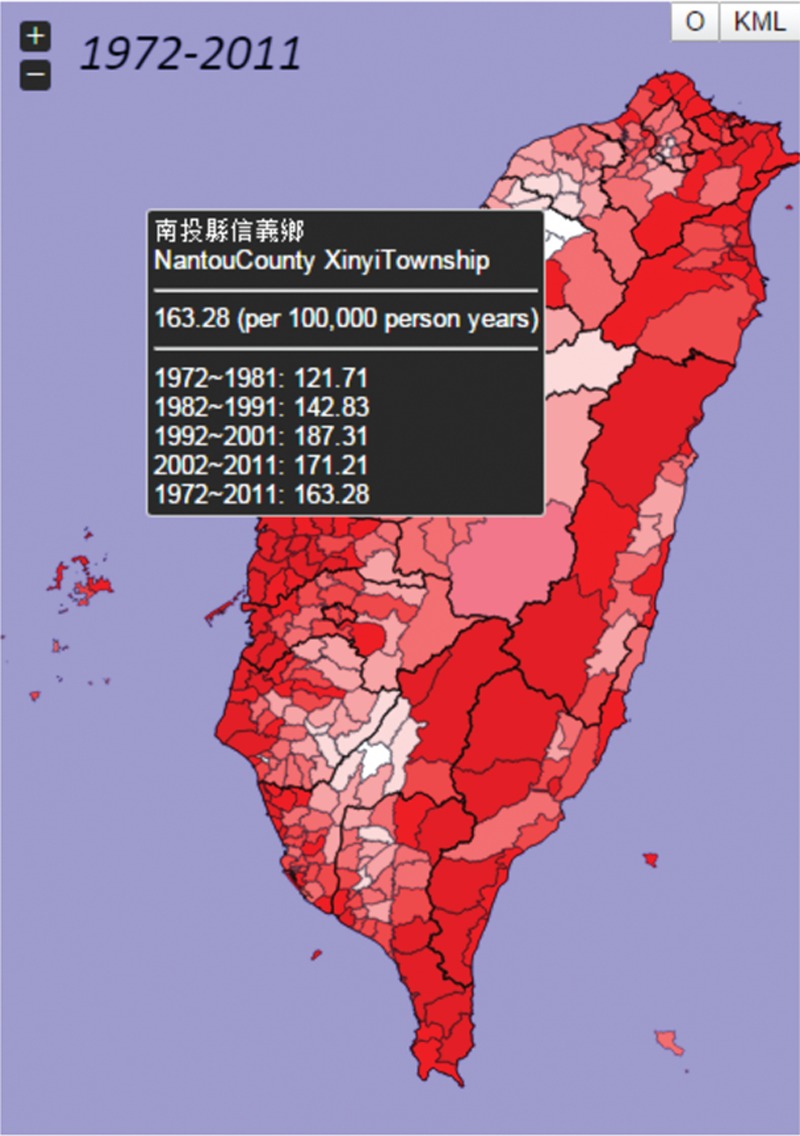
Example of an image map: jQuery tooltip for exploration of data by township.

**FIGURE 6:**
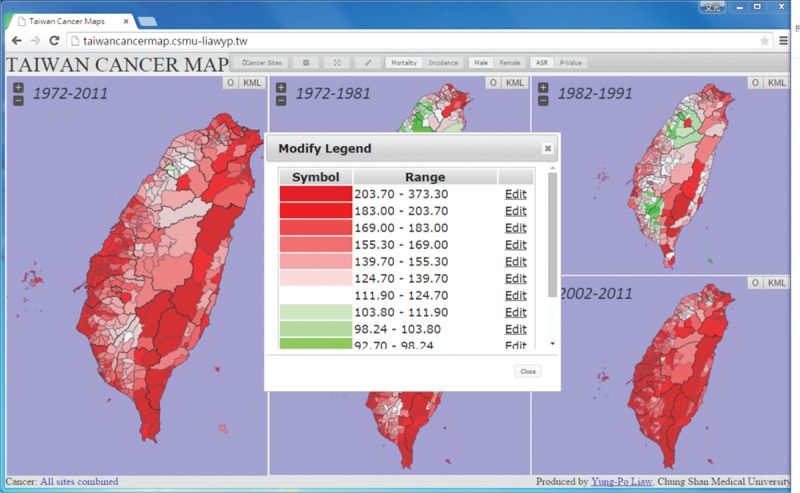
A screenshot of the “Modify Legend” which enables users to modify the data range.

Besides the mortality/incidence, gender, cancer type, and map index, the atlas also provides other basic functions and tooltip to enable exploration by township (Figure 5). It also provides a legend panel which enables users to adjust or modify map parameters at their convenience (Figure 6).

**FIGURE 7:**
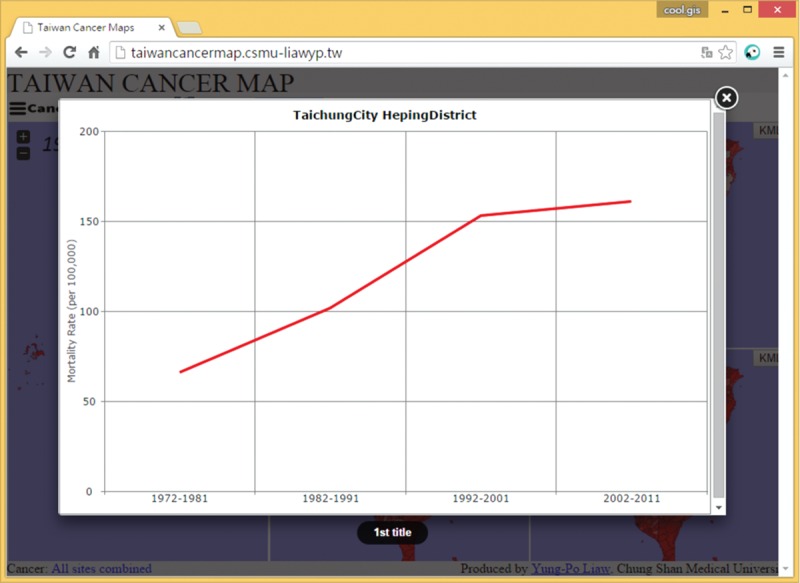
A screenshot of the display of “Chart” showing trends in cancer mortality in a township.

From the map, users can detect the changes in cancer mortality and incidence in Taiwan. The system provides chart tools that can show cancer trends for Individual Township (Figure 7).

**FIGURE 8:**
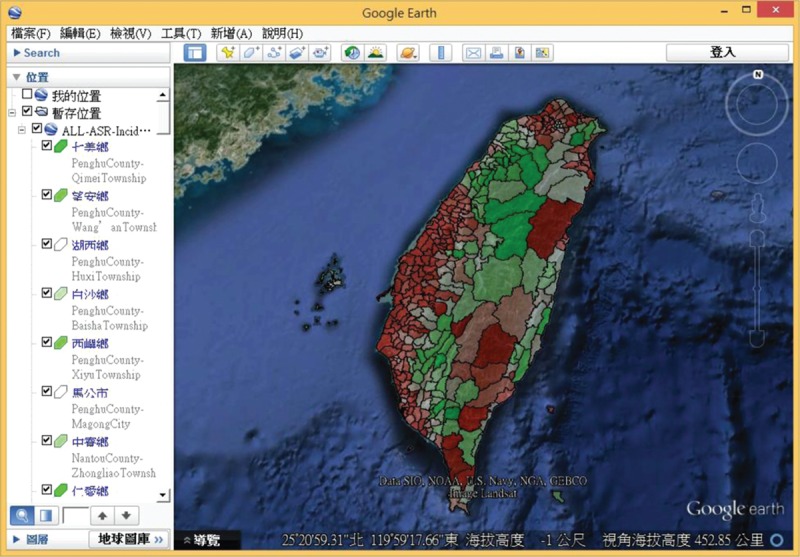
A screenshot of the default display of “Google Earth” which overlays a KML file (all township ASR). ASR = age-standardized mortality/incidence rate.

Google Earth is an easy-to-use visualization tool that allows users to explore geospatial information. The system provides an export function in which the cancer map can be exported to KML format and then overlay on Google Earth (Figure 8).

## DISCUSSION

To our knowledge, this is the first online atlas (with spatio-temporal cancer data) with a cross platform. In order to have access to the data, users simply need to type the following URL into their browser: http://taiwancancermap.csmu-liawyp.tw. It can be accessed through other devices such as personal computers, mobile phones, or pad. The platform is an effective, easy to use, updatable, and independent platform. The GIS can play an important role in generating hypotheses for many research projects. GIS is a powerful communication tool in public health which allows epidemiologists to investigate spatio-temporal patterns through visualization and analysis.^2,8,14–16^ However, most commercial GIS software are expensive and complex; hence, many researchers use GIS technologies free of charge in public health GIS analysis.^3,17–20^

This study shows that SVG combined with other web technologies (AJAX, JSON, JavaScript, jVectoryMap, jQuery) will minimize the amount of network transmission and enhance usage. This study uses open-source software such as AJAX, JSON, JavaScript, jQuery, SVG and jVectorMap free of charge, hence reducing development costs. With the development of the Internet, using the web for data mapping and sharing of public health information is becoming increasingly valuable and will be an important and exciting development trend ^21^ such as the WHO Global Health Observatory theme pages that also provide interactive map.^22^ WHO interactive maps use Adobe Flash. However, most of the mobile devices do not support the Flash plug-in and this hinders users from exploring the map. Relatively, our system is platform-independent and can render the map in a browser without requiring a plugin.

The application of SVG in public health can be traced back to 2005. Boulos and Russell constructed a diagnostic map of sexually transmitted diseases from 1997 to 2003 in London using SVG.^14^ Kamadjeu and Tolentino used SVG technology to develop a database-driven web-based GIS system using the EPI (Expanded Program on Immunization) data.^17^ However, early use of SVG as seen above was limited to web technologies. Each thematic map of cancer required to be pregenerated to an SVG file through the MapInfo software which, however, proved inconvenient. However, this was inconvenient. In this study, AJAX technology was used. The contents of the map are based on user settings which transfer data from the database server to the browser. In this way, the system has good scalability, flexibility, and a reduced maintenance cost. Furthermore, using AJAX technology can reduce network traffic and improve system performance to enhance user experience. This study made use of AJAX technology where the SVG model was generated only once before being reused on different thematic cancer maps. When a user selects cancer type, the system will send an AJAX request to the server. When the server receives this request, it will execute a database query and will respond to the browser in JSON format. Finally, the system will set the style of SVG element using JavaScript. By this way, it can render a new map by not having to leave the existing page. SVG with JavaScript is used to make maps more interactive and enables the user to explore the spatio-temporal health data. The system can also provide a function in which the cancer map can be exported in KML format and overlay on Google Earth. With Google Earth and its rich satellite images, users can assess the environment by region and this can help to generate research hypotheses.

Spatial autocorrelation is important to determine the risk factors for some specific types of cancer. However, the major aim of this study was not to investigate such factors. Mortality data were available from 1972 to 2011, whereas the incidence data were from 1995 to 2008. Aggregation of the data into shorter periods would produce unstable results. Moreover, dividing the data into longer periods would make it difficult to detect variation over time. Therefore, in this study, the mortality and incidence data were aggregated into 10 and 7-year periods, respectively.

This platform can be integrated with that of the WHO and can also be linked to Google Earth to generate hypotheses and to better comprehend cancer etiology. This technology can also make use of data from other countries. This will make it easier to compare cancer mortality and incidence patterns globally. Therefore, an easy to use, affordable, and independent online atlas is vital especially for those countries with high risk of cancer.

## CONCLUSION

This online atlas is a cheap and fast tool that integrates various cancer maps. Therefore, it can serve as a powerful tool that allows users to examine and compare spatio-temporal patterns of various maps.
